# Supplementary material for: Assessment of 1863 GRIN2A Variants Contradicts a Role in Tumorigenesis
Source: Int J Mol Sci. 2025 Jun 10;26(12):5558. doi: 10.3390/ijms26125558 (PMC12192978; doi:10.3390/ijms26125558)
Supplement: Supplementary file 1 [file ijms-26-05558-s001.zip › ijms-3647367 - Supplementary Captions.pdf]

Supplementary Table S1. *GRIN2A* variants identified in ClinVar, Cosmic and gnomAD.

Supplementary Table S2. List of tumor entity abbreviations based on TCGA nomenclature.

Supplementary Figure S1. Results of Kolmogorov–Smirnov test (testing for uniform variant distribution) visualised for all three datasets Cosmic (**A**), ClinVar (**B**) and gnomAD (**C**). Plots show observed variant distribution across cDNA (x-axis) versus expected uniform distribution. Value "D" gives the distance between observed and expected distribution, with high values indicating higher distance from expected uniform distribution. Somatic variants in the Cosmic dataset show only little distance from uniform distribution (D = 0.049).

Supplementary Figure S2. Boxplots of *GRIN2A* expression (log(TPM), y-axis) in tumor samples compared to non-disease samples (x-axis). No tumor tissue shows significantly higher expression of *GRIN2A* compared to the corresponding normal tissue. For tumor entity abbreviations see Supplementary Table S2.
