# Supplementary figures and images for: Assessment of 1863 GRIN2A Variants Contradicts a Role in Tumorigenesis
Source: Int J Mol Sci. 2025 Jun 10;26(12):5558. doi: 10.3390/ijms26125558 (PMC12192978; doi:10.3390/ijms26125558)

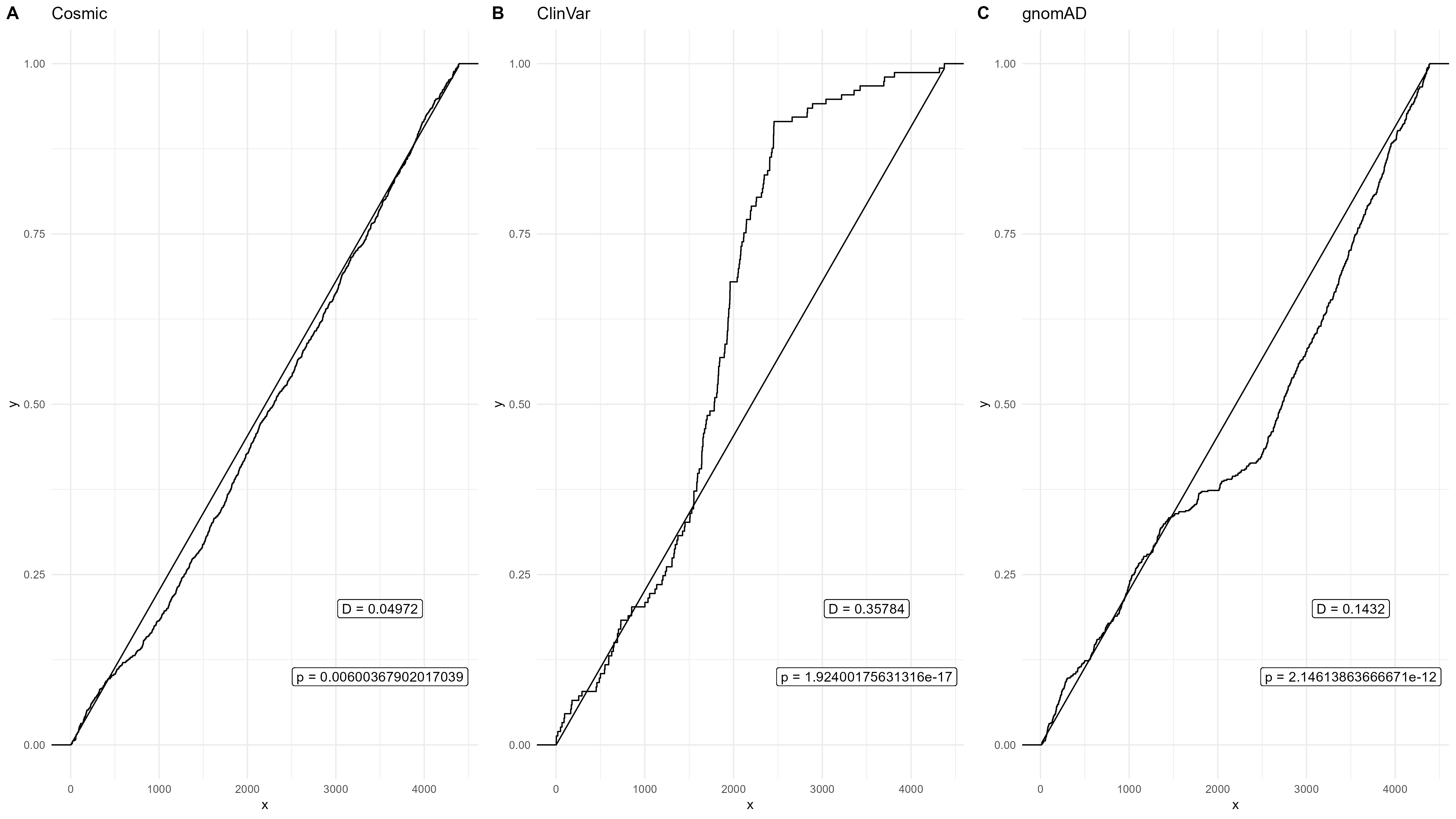

Supplement: Supplementary file 1 [file ijms-26-05558-s001.zip › Supplementary Figure S1.png]
